# Prevalence of prolonged QT interval in patients with HCV-related chronic liver disease

**DOI:** 10.1186/s43044-019-0016-0

**Published:** 2019-09-07

**Authors:** Ahmed E. Gaafar, Amr Abd El-Aal, Mohamed Alboraie, Housam M. Hassan, Adel ElTahan, Yasser AbdelRahman, Mohamed-Naguib Wifi, Dalia Omran, Shimaa Afify Mansour, Waleed M. Hassan, Magdy Ismail, Mohamed El Kassas

**Affiliations:** 1 Department of Cardiology, Faculty of Medicine, Helwan University, Mansour st., P.O. 11795 Ain Helwan, Cairo, Egypt; 2 Department of Internal Medicine, Faculty of Medicine, Al-Azhar University, Cairo, Egypt; 3 Department of Cardiology, Badr University Hospital, Helwan University, Cairo, Egypt; 4 New Cairo Viral Hepatitis Treatment Unite, Cairo, Egypt; 5 Department of Internal Medicine, Faculty of Medicine, Ain Shams University, Cairo, Egypt; 6 Department of Internal Medicine, Hepatogastroenterology unite, Cairo University, Cairo, Egypt; 7 Department of Endemic Medicine, Faculty of Medicine, Cairo University, Cairo, Egypt; 8 Faculty of Pharmaceutical Science, Cairo University, Cairo, Egypt; 9 Department of Endemic Medicine, Faculty of Medicine, Helwan University, Cairo, Egypt

**Keywords:** Hepatitis C virus, Direct-acting antivirals, QT prolongation, FIB-4 score

## Abstract

**Background:**

Hepatitis C virus (HCV) is a common disease in Egypt with a high socioeconomic burden and extra-hepatic manifestations as QT prolongation, but previous studies included mainly patients with advanced liver disease, so in this study, we aimed to delineate the prevalence of QT prolongation in early-stage HCV patients.

**Results:**

The study included 874 HCV patients with early cirrhosis; in Child’s class A, 57 (6.5%) patients had prolonged QT interval corrected (QTc). There was significant higher proportion of cirrhotic patients in the prolonged QTc group (31.6%) vs. in the normal QTc group (11.5%). QTc was 424.39 ± 36.6 vs. 411.51 ± 32.89 ms in cirrhotic and non-cirrhotic patients, respectively (*P*, 0.001). There was significant higher proportion of Fibrosis 4 (FIB-4) ≥ 1.45 score in the prolonged QTc (77.2%) vs. in the normal QTc group (56.8%) (*P*, 0.003). QTc interval was 417.76 ± 34.12 ms in patients with FIB-4 score ≥ 1.45 vs. 406.78 ± 31.95 ms in those with FIB-4 < 1.45 (*P*, < 0.001). FIB-4 score value of 2.108 predicted prolonged QTc with a sensitivity of 63.2% and a specificity of 64.5% (*P*, < 0.001). Twenty-four patients of long QTc group sent ECGs after HCV eradication, and 19 patients (79%) showed QTc normalization.

**Conclusions:**

HCV is associated with QTc prolongation even in patients with early chronic liver disease stages without significant fibrosis. Also, it is related to the degree of fibrosis and cirrhosis. At a cutoff value of 2.108, FIB-4 score can predict prolonged QTc. HCV eradication is associated with a high incidence of QTc normalization.

## Background

Hepatitis C virus (HCV) infection is considered as one of the most important global health problems; approximately, 177.5 million persons are considered infected [1]. Egypt faces the largest burden of HCV infection in the world which enforced the country to launch its large national control program [2]

Patients with HCV infection are at an increased cardiac risk. Many studies correlated the presence of HCV infection with larger intima-media thickness, higher prevalence of carotid plaques (3), transient ischemic attacks, and ischemic stroke [3, 4], and also, they had higher prevalence of coronary artery disease (CAD) [5], angina, and myocardial infarction [6]. However, the association between CAD and HCV remains unclear [7].

QT interval represents ventricular depolarization and repolarization. Prolonged QT interval leads to malignant ventricular tachyarrythmias [8]. This has a high impact on the patient’s activity, work, quality of life, life expectancy, and the economic burden it constitutes. Several studies have shown that cirrhosis is associated with prolonged QT interval [9–11]. It was found that HCV infection independently prolongs corrected QT interval, and its co-infection with human immunodeficiency virus increases the likelihood of clinically significant prolonged corrected QT interval [12]. In addition, patients with cirrhosis who had transplanted liver showed normalization of QT interval after liver transplantation, suggesting that this prolongation of QT interval is reversible [13, 14]. Published case reports documented that patients with prolonged QT interval have experienced repeated ventricular arrhythmias and significant hemodynamic instability during liver transplantation, which necessitates the preoperative recognition of prolonged QT interval and adequate treatment to prevent fatal arrhythmias [15].

In the past, it had been shown that the eradication of HCV with PEG-IFN plus ribavirin therapy caused an improvement of myocardial injury parameters in responders and even transient improvement in patients who suffered from relapse [16]. Now, the evolution of direct-acting drugs (DAAs) such as sofosbuvir, simeprevir, daclatasvir, ledipasvir, and others has changed the standard of care to different combination therapy of DAAs with excellent results and adequate safety profile [17].

Despite the high prevalence of chronic HCV infection in the Egyptian community and the increasing availability of DAAs, there is a lack of data regarding the use of DAAs with associated HCV cardiovascular risks. In this study, we aimed to estimate the prevalence of prolonged QT interval in Egyptian patients with chronic HCV in early stages and the effect of HCV eradication with DAAs on prolonged QT interval.

## Methods

### Patients’ selection

Our study included 874 patients attending specialized HCV outpatient clinics of New Cairo Viral Hepatitis Treatment Center (NCVHTC), with a diagnosis of chronic HCV, based on the polymerase chain reaction (PCR) positivity for HCV RNA, between January 2015 and October 2017. An age- and sex-matched control group of apparently healthy 107 subjects were used to compare their QT interval corrected (QTc) with our study group. All patients presented with chronic hepatitis; in Child’s class A (the score is calculated based on five parameters (total bilirubin level, serum albumin, international normalized ratio, degree of ascites, and degree of hepatic encephalopathy), it became widely used to predict the prognosis in patients with chronic liver disease and cirrhosis [18, 19]. Any patient with suspected cardiac abnormalities during pre-treatment assessment by the hepatologists was referred to the cardiologist for reassessment. Baseline ECG was performed for all subjects before initiation of any direct antiviral agents as necessitated by the standardized HCV treatment protocol. For those patients with more than one ECG available before antiviral therapy, only the most recent was used for analysis. All patients were subjected to laboratory studies: serum alanine aminotransferase (ALT), aspartate aminotransferase (AST), total and direct bilirubin, serum albumin, prothrombin time and international normalization ratio (PT, INR), fasting blood sugar and HbA1C for diabetic patients, serum creatinine, alpha-fetoprotein (AFP), complete blood count, and thyroid-stimulating hormone (TSH). Abdominal ultrasound and fibroscan were done for all patients in addition to Fibrosis 4 (FIB-4) score calculation which is a panel of routine blood tests (ALT, AST, and platelet count) in addition to age that is used in an equation [20] to predict the stage of hepatic fibrosis. FIB-4 score < 1.45 had a negative predictive value of 90% for advanced fibrosis (Ishak fibrosis score 4–6). Exclusion criteria included any other known causes of long QT interval as administration of drugs that may affect long QT interval (as beta-blockers as it can mask QT prolongation, diuretics as it can alter electrolyte levels, antiarrhythmic drugs … etc.), jcc3001, electrolyte abnormalities as hypokalemia (˂ 3.5 meq/L) and hypomagnesaemia (˂ 1.5 meq/L), known or suspected structural heart disease as ischemic heart disease, heart failure (by history, symptoms, echocardiography or ECG suggestive of CAD or myocardial disease as left ventricular hypertrophy), congenital heart disease, significant valvular heart disease and presence of any significant arrhythmias (frequent PVCs, atrial fibrillation, heart block, interventricular conduction delay, or bundle branch blocks), or family history of long QT syndrome or sudden cardiac death with suspected long QT syndrome. Furthermore, patients who are not eligible for HCV antiviral therapy as in Child’s C cirrhotic patients, platelet count ≤ 50,000/mm, and those with pregnancy or inability to use effective contraception were excluded.

### Determination of QT interval

QT interval was read from a 12-lead electrocardiogram recorded at 50 mm/s. QT interval was measured from the beginning of the QRS complex until the termination of the T wave. The QT interval corrected for heart rate (QTc) was measured in limb lead II (or lead I or III if it could not be measured exactly in lead II) with the use of Bazett’s formula. QTc intervals were averaged from five consecutive beats. Since heart rate was a major determinant of the duration of ventricular repolarization, QT interval had to be corrected for the heart rate. In this study, QTc (QT interval corrected for heart rate) was calculated as the ratio of the calculated QT interval in seconds to the square root of RR interval in seconds (standard Bazett’s formula) [21]. QTc interval ≥ 450 ms (millisecond) for males and ≥ 470 ms for females was considered prolonged QTc cutoff values.

The QTc calculation was done blindly and manually by expert cardiologists and compared with the values calculated automatically by the ECG machine to avoid errors due to ECG artifacts.

### HCV antiviral regimens

All patients were treated according to the protocol of the National Committee for Control of Viral Hepatitis (NCCVH) with either a combination of sofosbuvir and simeprevir or a combination of sofosbuvir and daclatasvir for 3 months

### Treatment monitoring

All patients were subjected to PCR testing at week 12 post-treatment to confirm successful eradication of the virus.

### Follow-up

Patients who had prolonged QTc were asked to perform ECG after eradication of HCV to detect the effect of HCV eradication on QT interval

### Statistical analysis

Patients with prolonged QTc and those with normal QTc were compared for binary variables with the chi-square test. The independent *t* test was used for continuous variables after assessing the equality of variance using Levene’s test. If a statistically significant difference was found in the proportion of FIB-4 groups between prolonged QTc and normal QTc groups, ROC analysis will be done to determine the cutoff of FIB-4 score that may predict prolonged QTc. The best cutoff point will then be determined according to the point which gives the highest sensitivity plus specificity. Statistics were computed using Statistical Package for Social Science (IBM SPSS) version 20. All tests were two-tailed with the significance level set at 0.05.

## Results

The study included 986 patients, 874 patients were included and 112 were excluded (beta-blocker use in 47 patients, abnormal ECG in 16 patients, AF in 7 patients, other arrhythmias in 6 patients, hypokalemia in 2 patients, CAD in 11 patients, QTc active drugs in 3 patients, valvular heart disease in 9 patients, left ventricular hypertrophy by echocardiography in 7 patients, heart failure in 4 patients) Among 874 chronic HCV patients, 57 (6.5%) patients showed prolonged QTc which was significantly higher incidence in comparison to the control group where it was found in one subject (0.9%) (*P* value < 0.05). The studied patients were divided into two groups: patients with prolonged QTC and patients with normal QTc. Patient characteristics for the studied population were demonstrated in a table (Table 1). There was a statistically significant older age in the group with prolonged QTc (53.7 ± 9.8 years vs. 50.1 ± 11.6 years in normal QTC group, *P* value = 0.024). Also, hypertension (HTN) was significantly more prevalent in the prolonged QTc group (31.6 % vs. 16.8% in normal QTc group, *P* value = 0.005). There was a marginally statistically significant difference in the tobacco consumption between both groups (7% in prolonged QTc group vs. 17.4% in normal QTc group, *P* value = 0.043). There was no statistically significant difference between the prolonged QTc group and normal QTc group for all laboratory tests except serum albumin (3.84 ± 0.50 g/dL in prolonged QTc group vs. 4.00 ± 0.49 g/dl in normal QTc group, *P* value = 0.013) (Table 2).

**Table 1:** Baseline characteristics of the studied patients

Parameter		Long (QTc)	Normal (QTc)	*P* value
No. (%)		57 (6.5%)	817(93.5%)	
Age		53.7 ± 9.8	50.2 ± 11.6	0.024*
Gender	Male	33 (57.9%)	406 (49.7%)	0.273
	Female	24 (42.1%)	411 (50.3%)	
Tobacco consumption		4 (7.0%)	142 (17.4%)	0.043*
BP		131 ± 19	125 ± 21	NS
Hypertension		18 (31.6%)	137 (16.8%)	0.005*
DM		19 (33.3%)	196 (24.0%)	0.113
HR		83 ± 14	79 ± 18	NS

*DM* diabetes mellitus, * for signeficant data

**Table 2:** The laboratory tests done for the studied patients

Parameter	Long (QTc)	Normal (QTc)	*P* value
No. (%)	57 (6.5%)	817 (93.5%)	
ALT (IU/L)	65.228 ± 73.16	50.909 ± 36.045	0.148*
AST (IU/L)	60.632 ± 50.03	47.991 ± 32.440	0.065*
AFP (IU/L)	13.732 ± 22.47	10.678 ± 52.591	0.666
Serum albumin (g/dL)	3.837 ± 0.5	4.003 ± 0.488	0.013
TSH (μIU/mL)	1.933 ± 1.25	1.897 ± 1.263	0.896
Total serum bilirubin (mg/dL)	0.916 ± 0.45	0.796 ± 0.374	0.210
Creatinine (mg/dL)	0.840 ± 0.25	0.854 ± 0.257	0.694
Viral quantity (PCR)	1,391,605. ± 1660179.6	1,524,056.4 ± 2,173,335.63	0.655
ANC × 10^3^/mm^3^	3.175 ± 1.600	3.374 ± 1.494	0.343

.*ALT* alanine aminotransferase, *AST* aspartate aminotransferase, *AFP* alpha-fetoproteins, *TSH* thyroid-stimulating hormone, *ANC × 10*^*3*^*/mm*^*3*^ = neutrophil count

*Equal variance not assumed

Liver cirrhosis was significantly higher in prolonged QTc group (31.6% vs. 11.5% in normal QTc group, *P* value = <0.005). Mean QTc interval was 424.39 ± 36.6 ms in cirrhotic patients vs. 411.51 ± 32.89 ms in non-cirrhotic ones (*P* value = 0.001). Moreover, on categorizing patients according to FIB-4 score into two groups: patients with a FIB-4 score ≥ 1.45 (FIB-4 suggesting advanced fibrosis) and patients with FIB-4 < 1.45 (suggesting an absence of advanced fibrosis). We found that there was statistically significant higher proportion of FIB-4 ≥ 1.45 score in the prolonged QTc group (77.2%) vs. 56.8 % in normal QTc group (*P* value = 0.003) (Table 3). The mean QTc interval was 417.76 ± 34.12 ms in patients with FIB-4 score ≥ 1.45 vs. 406.78 ± 31.95 ms in FIB-4 < 1.45( *P* value < 0.001). ROC analysis for FIB-4 score revealed that at 2.108, FIB-4 was able to predict prolonged QTc with a sensitivity of 63.2% and a specificity of 64.5% (*P*, < 0.001). The area under the curve was 0.645. Multivariate analysis showed that age and hypertension were independently associated with prolonged QT interval (Table 4).

**Table 3:** Prevalence of long QT interval in cirrhotic patients and in patients with significant fibrosis (FIB-4 ≥ 1.45)

		Long (QTc)	Normal (QTc)	*P* value
No. (%)		57 (6.5%)	817 (93.5%)	
Liver status	Cirrhotic liver	18 (31.6%)	94 (11.5%)	< 0.005
FIB-4 score	> 1.45	44 (77.2%)	464 (56.8%)	0.003

*QTc* corrected QT interval for heart rate using Bazett’s formula

**Table 4:** Multivariate analysis for factors associated with long QT

Model	Odds ratio	*P* value	95% CI
Constant	411.371	< 0.001	386.06–436.681
Age	0.381	< 0.001	0.168–0.594
Tobacco consumption	− 16.572	< 0.001	− 22.344 to − 10.799
Hypertension	6.616	0.027	0.75–12.482
Serum albumin (g/dL)	− 3.304	0.171	− 8.035–1.427
Liver cirrhosis	4.257	0.215	− 2.482–10.995
FIB-4 score	1.068	0.059	− 0.04–2.176

Liver cirrhosis was diagnosed by abdominal ultrasound examination

Of the 57 patients with prolonged QTc, only 24 patients did a follow-up ECG after completing an antiviral regimen, 19 patients (79%) showed normalization of QTc while 5 patients (21%) did not show QTc normalizations. All of the 24 patients were responders to the antiviral treatment given with documented eradication of HCV by PCR.

## Discussion

The duration of the QT interval calculated by 12-lead electrocardiography is a measure of ventricular repolarization. Abnormalities of ventricular repolarization may provide the substrate for ventricular arrhythmia and sudden death, and thus, it is a vital component of both ECG diagnostics and medical decision-making. Several acquired and congenital cardiac conditions as well as electrolyte disturbance and widely used drugs are associated with QT interval prolongation [22]. Initially, prolonged QT interval and sudden deaths were identified in patients with alcoholic cirrhosis and then it was elaborated in cirrhosis of different etiologies along with the severity of disease, portal hypertension, development of portosystemic shunts, and decreased survival [23]. However, prolonged QTc interval can also be seen in well-compensated liver disease [24].

Most patients in our study had an early stage of HCV-related chronic liver disease. This may explain the relatively low prevalence of prolonged QT interval in comparison to other studies including Child’s B and C patients. Prolongation of QT interval was correlated with advanced cirrhosis [25–30]. Also, we used higher QTc cutoff values (450 ms for males and 470 ms for females) while most of the previous studies used 440 ms as a cutoff value irrespective to the known gender differences; moreover, these studies had fewer numbers of patient ranging from 50 to 200 patients. So we presume that our study has a more specific correlation between HCV and QTc prolongation and higher specificity to detect pathological QTc prolongation. Incidence of long QTc in our study was similar to Bernardi et al. who reported a QT prolongation prevalence rate of 5.4% in patients with chronic HCV [31].

Although few patients had follow-up electrocardiogram after HCV eradication, yet 79% of patients with prolonged QTc showed QTc normalization after HCV eradication, despite this small sample size that hinders statistical analysis, this figure gives a clear trend that supports the direct relation between HCV and prolonged QTc. The normalization of QTc in these patients cannot be attributed to the improvement of liver functions or clinical status because the studied group had normal lab from the start and were classified as Child’s A liver disease; also, the variation of heart rate before and after viral eradication is unlikely to be a cause as we used a heart rate-corrected QT interval to abolish the rate variability effect and we did not use the QT interval. Moreover, our study revealed that the prevalence of long QT interval increased significantly in the presence of advanced liver fibrosis and in advanced cirrhosis compared to non-cirrhotic and non-fibrosed patients.

In the current study, patients with prolonged QT were associated with lower albumin level than normal QT group. Although this result is concordant with previous studies that related QT interval prolongation with the severity of liver disease [25, 27, 29], yet lower serum albumin level in prolonged QTc group is unlikely to be the cause of QTc prolongation as the albumin levels in both groups are still within normal values. Genovesi et al. showed a significant correlation between liver cirrhosis stage according to Child-Pugh classification and QT interval prolongation [26]. Another study revealed a correlation between the QT interval prolongation and the severity of cirrhosis but not the cirrhosis etiology [32]. Bal and Thuluvath reported that prolonged QT interval was common in cirrhosis but did not influence the mortality rate [33]. On the contrary, other studies found no correlation between QT interval prolongation and the severity of cirrhosis [34–36].

The mechanism responsible for the prolongation of QT interval is unknown. Alterations at a molecular level had been suggested [30]. Other factors include electrolyte abnormalities, myocardial ischemia, and alterations in the autonomic nervous activity that might influence the heart rate and electromechanical coupling by several mechanisms [23, 28, 37]. Abnormalities in gonadal hormone metabolism that occurs in advanced cirrhosis had been suggested to contribute to the development of QT prolongation in this setting [38, 39]. In the current study, we found that QTc interval was significantly longer in females. It had been reported that females are more prone to torsade de pointes than males which was correlated to the quantitative gender difference in electrocardiogram manifestation of myocardial repolarization. This sex disparity has been confirmed and applied to different electrocardiogram indices of myocardial repolarization duration [40–42].

Our study showed that hypertension (HTN) was an independent predictor of QT prolongation in the patient with HCV-related chronic liver disease. The mechanism of QT prolongation with HTN is not well understood, and it is mostly multifactorial and is related to changes in left ventricular transmural dispersion of repolarization accompanying myocardial cells hypertrophy and increased left ventricular mass in addition to the disturbance in the tone of the autonomic nervous system and mechanoelectrical feedback [43]. But in our study, we think that HTN has an insignificant contribution in QTc prolongation as there were no significant LVH signs in the ECGs of hypertension patients which exclude significant LVH or uncontrolled hypertension and that is why we excluded patients with ECGs suggestive of myocardial diseases. Concordant to our study, Reardon and Malik reported that in an apparently normal population, QTc increased with age and this might be due to fibrosis of the myocardium as part of the aging process affecting the heart together with the imbalance between sympathetic and parasympathetic tone seen with aging leading to an exaggerated swing towards sympathetic activity. Moreover, HCV was reported to cause myocardial fibrosis by itself [43]. Omran et al reported that myocardial fibrosis was detected in 83% of their HCV patients with a mean of 19% of myocardial mass [44].

Our study was the first (to our knowledge) that presents a cutoff value for FIB-4 score of 2.108 at which this can predict prolonged QTc with a sensitivity of 63.2% and a specificity of 64.5% and this may be of prognostic significance since prolonged QTc interval is associated with a high risk of sudden death [32, 43].

## Limitations

Patient adherence to follow up was poor which lead to longer than expected duration for recruitment of our sample size, and it was assumed to be due to the socioeconomic factors. We did not examine the ECGs of the patients during the treatment period as we design the study to explore the incidence of QTc prolongation in the studied group. We did not test all patients for electrolyte levels and did only for patients with a risk for disturbance as those with renal impairment or steroid therapy. Duration of HCV infection was not known as most patients were discovered during the national screening program. The type of antiviral drug used in each group was not included in the comparison. We did not perform inter- or intra-observer variation as regard ECG interpretation.

## Conclusion

Prolonged QT interval is present in patients with chronic HCV and early cirrhosis. So it is important to follow QT interval even in patients in early cirrhosis. However, it is related to the stage of hepatic fibrosis, being significantly more prolonged in those with advanced fibrosis and cirrhosis. Old age and hypertension are independent factors associated with the prolongation of QTc interval. At a value of 2.108 FIB-4 score, we recommend a closer follow-up of QTc.

## Data Availability

The datasets used and/or analyzed during the current study are available from the corresponding author on reasonable request.

## Abbreviations

AFP: Alpha-fetoprotein
ALT: Serum alanine aminotransferase
AST: Aspartate aminotransferase
CAD: Coronary artery disease
DAAs: Direct-acting antiviral drugs
FIB-4 score: Fibrosis 4 score
HbA1: Glycated hemoglobin
HCV: Hepatitis C virus
HTN: Hypertension
INR: International normalization ratio
LVH: Left ventricular hypertrophy
PCR: Polymerase chain reaction
PT: Prothrombin time
PVCs: Premature ventricular contractions
TSH: Thyroid-stimulating hormone
