# Correction to: Prevalence of prolonged QT interval in patients with HCV-related chronic liver disease

**DOI:** 10.1186/s43044-019-0020-4

**Published:** 2019-10-29

**Authors:** Ahmed E. Gaafar, Amr Abd El-Aal, Mohamed Alboraie, Housam M. Hassan, Adel ElTahan, Yasser AbdelRahman, Mohamed-Naguib Wifi, Dalia Omran, Shimaa Afify Mansour, Waleed M. Hassan, Magdy Ismail, Mohamed El Kassas

**Affiliations:** 1 Department of Cardiology, Faculty of Medicine, Helwan University, Mansour st., P.O. 11795 Ain Helwan, Cairo, Egypt; 2 Department of Internal Medicine, Faculty of Medicine, Al-Azhar University, Cairo, Egypt; 3 Department of Cardiology, Badr University Hospital, Helwan University, Cairo, Egypt; 4 New Cairo Viral Hepatitis Treatment Unite, Cairo, Egypt; 5 Department of Internal Medicine, Faculty of Medicine, Ain Shams University, Cairo, Egypt; 6 Department of Internal Medicine, Hepatogastroenterology unite, Cairo, University, Cairo, Egypt; 7 Department of Endemic Medicine, Faculty of Medicine, Cairo University, Cairo, Egypt; 8 Faculty of Pharmaceutical Science, Cairo University, Cairo, Egypt; 9 Department of Endemic Medicine, Faculty of Medicine, Helwan University, Cairo, Egypt

**Correction to: Egypt Heart J (2019) 71:15**

**https://doi.org/10.1186/s43044-019-0016-0**

Following publication of the original article [1], the authors reported that the family name of Mohamed El Kassas was incorrectly published as Mohamed ElKassas.

The original article [1] has been corrected.
